# Pre-existing humoral immunity in military smallpox vaccinees temporally effects MVA-vectored transgene expression in dendritic cells

**DOI:** 10.1186/1742-4690-9-S2-P97

**Published:** 2012-09-13

**Authors:** V Ngauy, B Slike, M Marovich

**Affiliations:** 1 Armed Forces Research Institute of Medical Sciences, Bangkok, Thailand; 2 US Military HIV Research Program, MD, USA

## Background

Modified Vaccinia Ankara (MVA), a vector-based vaccine that targets dendritic cells (DC) and induces cell-mediated immunity, is a promising HIV vaccine candidate. As MVA vaccination strategies continue to be explored, concerns arise regarding transferability to individuals with pre-existing immunity to vaccina, particularly military personnel. Prior reports suggest long-lasting vaccinia immunity after childhood vaccination. This study, conducted in a unique cohort of adult primary vaccinees, explores how pre-exisiting humoral immunity to vaccinia affects entry and transgene expression of MVA-vectored vaccines in a primary human DC infection model.

## Methods

Serum from military personnel vaccinated against smallpox with either Dryvax or ACAM2000 vaccines were obtained at 4 time points post-vaccination (n=50 per time point, 400 total). As a comparator, n=25 individuals with longitudinal sera available at corresponding time points were studied to compensate for inter-individual variability in response over time. Sera were tested for inhibition of infection of DCs in vitro using either MVA-GFP or MVA-CMDR, an HIV vaccine candidate with env/gag/pol inserts, currently in clinical trials. Vaccinia naïve sera served as a negative control. Vaccinia binding titers were measured by ELISA.

## Results

Vaccinia binding antibody titers waned after 5 years and were undetectable 10 years after vaccination. Neutralizing activity, as measured by transgene expression in DCs, confirmed this finding. Expression and neutralization of HIV p24 (gag) expression data in DC were equivalent to that of GFP. No differences in neutralization activity were detected between Dryvax and ACAM2000 vaccinee sera at corresponding time points.

## Conclusion

In an adult, military, primary vaccinee population, humoral responses to smallpox vaccination do not persist as long as reported in the civilian population vaccinated during childhood. This data suggests that pox-vector based vaccines may be used in the military population, and that the age of primary vaccination influences durability of humoral immunity.

